# Dynamical assessment of fluorescent probes mobility in poplar cell walls reveals nanopores govern saccharification

**DOI:** 10.1186/s13068-018-1267-9

**Published:** 2018-10-03

**Authors:** Mickaël Herbaut, Aya Zoghlami, Gabriel Paës

**Affiliations:** Fractionation of AgroResources and Environment (FARE) Laboratory, INRA, University of Reims Champagne-Ardenne, Reims, France

**Keywords:** Biomass, Pretreatment, Saccharification, PEG-rhodamine, FRAP, Accessibility, Porosity

## Abstract

**Background:**

Improving lignocellulolytic enzymes’ diffusion and accessibility to their substrate in the plant cell walls is recognised as a critical issue for optimising saccharification. Although many chemical features are considered as detrimental to saccharification, enzymes’ dynamics within the cell walls remains poorly explored and understood. To address this issue, poplar fragments were submitted to hot water and ionic liquid pretreatments selected for their contrasted effects on both the structure and composition of lignocellulose. In addition to chemical composition and porosity analyses, the diffusion of polyethylene glycol probes of different sizes was measured at three different time points during the saccharification.

**Results:**

Probes’ diffusion was mainly affected by probes size and pretreatments but only slightly by saccharification time. This means that, despite the removal of polysaccharides during saccharification, diffusion of probes was not improved since they became hindered by changes in lignin conformation, whose relative amount increased over time. Porosity measurements showed that probes’ diffusion was highly correlated with the amount of pores having a diameter at least five times the size of the probes. Testing the relationship with saccharification demonstrated that accessibility of 1.3–1.7-nm radius probes measured by FRAP on non-hydrolysed samples was highly correlated with poplar digestibility together with the measurement of initial porosity on the range 5–20 nm.

**Conclusion:**

Mobility measurements performed before hydrolysis can serve to explain and even predict saccharification with accuracy. The discrepancy observed between probes’ size and pores’ diameters to explain accessibility is likely due to biomass features such as lignin content and composition that prevent probes’ diffusion through non-specific interactions probably leading to pores’ entanglements.

## Background

Lignocellulosic biomass such as dedicated crops or agricultural and wood residues is one of the most abundant yet under-utilised bioresources in the world and offers substantial possibilities to overcome our reliance on fossil carbon resources [1–3]. Cellulose, hemicelluloses and lignin contained in the plant cell walls can be depolymerised to produce a large range of compounds such as biofuels, fibres, plastics or chemicals that are currently industrially produced through petrochemical processes [4, 5]. However, both composition and structural layout of the plant cell walls hamper lignocellulolytic enzymes progression and activity, making biomass recalcitrant to enzymatic hydrolysis [6]. Pretreatments are mandatory steps prior to saccharification to disorganise the plant cell walls structure, and to improve enzymes access and activity to their substrate, thus optimising the conversion of lignocellulose [5, 7]. Hot water (HW) pretreatment is considered as an environment-friendly pretreatment process which allows removing hemicelluloses that can be extracted as valuable xylo-oligosaccharides. Lignin can undergo depolymerisation and condensation reactions modifying its distribution within the plant cell wall, but the remaining lignin can sometimes impact the enzymatic degradation of cellulose [8]. Ionic liquids (ILs) are promising green solvents allowing the dissolution of lignocellulose in relatively mild conditions, partially removing lignin and making cellulose more amorphous and easier to hydrolyse [9, 10]. Among many ILs used to pretreat biomass samples, 1-ethyl-3-methylimidazolium acetate has shown good abilities in dissolving large biomass particles compared to other imidazolium-based ILs [11].

Several compositional and structural plant cell walls’ features are recognised as factors impacting saccharification independent of biomass species and pretreatment [6]. Lignin content, composition and structure strongly limit biomass deconstruction both by restricting the access to the polysaccharides and by non-productively binding enzymes [12, 13]. Lignin removal from the plant cell wall is likely to increase its global porosity, which also influences enzymes’ diffusion and is possibly directly correlated with biomass initial digestibility [14]. These factors influence cellulose accessible surface area which is also an important parameter as it governs enzymes binding to substrate [15, 16].

Different techniques can be used to assess lignocellulose accessibility through porosity measurements such as solute exclusion [17], Simon’s stain method [15, 18], nitrogen adsorption [19, 20], low-field nuclear magnetic resonance (NMR) spectroscopy [21, 22] or electron tomography [23]. However, these techniques only give an overview of structural changes that could potentially affect enzymes’ diffusion and binding to their substrate, and do not directly assess enzymes’ dynamics that could be impacted by other features such as interactions with lignin. The use of confocal laser scanning microscopy (CLSM) techniques can allow measuring enzymes’ behaviour inside the plant cell walls by direct observations of fluorescently labelled enzymes or fluorescent probes. Fluorescence Recovery After Photobleaching (FRAP) technique was recently used to assess the diffusion behaviour of dextrans and labelled cellulases in plant cell wall bioinspired assemblies [24, 25]. Mobility of *Bacillus subtilis* xylanases on wheat flour arabinoxylan and fluorescent probes inside pretreated poplar samples were also investigated directly using the FRAP technique [26, 27].

Another interesting probes that can be used to study the structure of the plant cell wall are polyethylene glycol (PEG) fluorescent probes. PEG probes are obtained by chemically linking ethylene glycol subunits, allowing the synthesis of a linear polymer of a specific size [28]. A fluorescent dye such as a rhodamine dye can then be grafted to one of the terminal hydroxyl groups of the polymer. PEG-rhodamine probes were selected as PEG was demonstrated to bind lignin through hydrophobic interactions, thus preventing unproductive binding of enzymes. PEG, thus, allows an increase in the glucose yield when added to a saccharification reaction medium [29, 30]. Donaldson et al. measured the interactions between PEG-rhodamine probes and lignin derived from the secondary walls of steam-exploded wood samples chips using CLSM and showed that these interactions were responsible for an increase of the glucose yield when the PEG-rhodamine was added to the saccharification medium [31]. PEG-rhodamine probes, hence, mimic the non-specific interactions of proteins with lignin while inducing no chemical modification of the cell wall due to the absence of catalytic activity, making them ideal probes to assess enzyme dynamics in cell walls.

In this study, we have used CLSM to assess the dynamic behaviour of rhodamine-labelled PEG probes in untreated and pretreated poplar fragments, to explore lignocellulose accessibility. First, the effect of the different pretreatments on poplar fragments’ porosity and enzymatic hydrolysis was measured. Then, hydrolysed poplar fragments were collected at different time points during the saccharification, thoroughly washed and incubated with PEG-rhodamine probes of different sizes so that their composition and accessibility measured by FRAP were assayed over time. The influence of the pretreatments, the probes’ size and the porosity was calculated to determine which parameter influences enzyme dynamics during the saccharification.

## Methods

### Plant materials

Poplar (*Populus nigra* × *deltoides*) was cultivated on experimental plots in Estrées-Mons (France) and harvested 2 years after planting. Fragments 2 × 0.6 × 0.2 cm in size were cut from branches using a razor blade. Ramification regions were put aside to avoid tension wood.

### Pretreatments

#### Hot water pretreatment

Hot water (HW) pretreatment was performed on biomass fragments using deionised water at a ratio of 1:30 (500 mg of biomass for 15 mL of deionised water). Pretreatments were performed using mineralisation bombs equipped with Teflon cups (Parr). Samples were kept at 180 °C for 60 min in an oil bath. The fragments were then cooled down in ice and thoroughly washed in deionised water and 50% ethanol.

#### Ionic liquid pretreatment

Ionic liquid (IL) pretreatment was performed on biomass fragments using 1-ethyl-3-methylimidazolium acetate (Solvionic, France) with a biomass loading of 6% (w/v). Pretreatments were performed using mineralisation bombs equipped with Teflon cups (Parr). Samples were kept at 130 °C for 40 min in an oil bath. The fragments were then cooled down in ice, regenerated in deionised water at 4 °C, filtered using 20 volumes of deionised water and thoroughly washed in deionised water and 50% ethanol to remove any ionic liquid.

### Enzymatic hydrolysis

Saccharification experiments were performed using the Cellic ^®^ CTec 2, cocktail kindly provided by Novozymes (Denmark), whose activity was determined to be 157 FPU/mL according to the filter paper method using Whatmann no 1 filter paper as standard substrate [32]. The cellulase cocktail was used in a 0.1 M citrate buffer at pH 4.8 containing 0.02% sodium azide with a biomass loading of 2% (w/v) and an enzyme loading of 90 FPU/g of biomass. Experiments were stopped after different saccharification durations (0 h, 15 h and 96 h) and fragments were recovered from the reaction medium and thoroughly washed with deionised water for further analyses. The glucose released in the reaction medium over time was measured by anionic exchange chromatography as previously described [13].

### Polysaccharides analysis

The overall fragments sugar content was assessed by acid hydrolysis as previously described [33]. Samples collected after 0 h, 15 h and 96 h of enzymatic hydrolysis were milled to a granulometry of 80 µm and then submitted to a two-step H_2_SO_4_ hydrolysis: 125 µL of a 12M H_2_SO_4_ solution was added to 10 mg of biomass samples for 2 h at room temperature under stirring, then acid was diluted to 1 M for another 2-h incubation at 100 °C. Hydrolysed monomeric sugars were quantified by HPAEC–PAD [13].

### Klason lignin quantification

The acid-insoluble lignin content of the samples collected after 0 h, 15 h and 96 h of enzymatic hydrolysis was gravimetrically quantified by the Klason method as previously described [34].

### Nitrogen content determination

Fragments collected at 0 h, 15 h and 96 h of enzymatic hydrolysis were milled to a granulometry of 80 µm before being oven-dried overnight at 80 °C, and 5–7 mg were weighted in tin capsule. Capsules were analysed using a EURO EA elemental analyser (Eurovector, Milan, Italy) equipped with a thermal conductivity detector. The samples were fully oxidised and nitrogen was converted into N_2_ and quantified using the Eager 200 software (Carlo Erba, Italy). Protein amount in the plant cell walls was then calculated by applying a nitrogen–protein conversion factor of ×6.25 [35].

### Porosity measurements

Fragments’ porosity was assessed by low-field nuclear magnetic resonance (LF-NMR) relaxation measurements. 1-cm-long biomass fragments were soaked in water for 96 h prior to LF-NMR analysis using a Minispec mq20 spectrometer (Bruker) as previously described [13].

### Probes’s hydrodynamic radius measurements

Methoxypolyethylene glycol molecules with an average MW of 5, 10 and 20 kDa and labelled with methylrhodamine were purchased from CreativePEGWorks (Chapel Hill, USA). The hydrodynamic radius of the fluorescent probes was measured by dynamic light scattering (DLS). DLS experiments were carried out using a system equipped with a DU 4007 degasser (UNIFLOWS Co., Japan), a WATERS 717 Plus Autosampler, a WATERS 515 HPLC pump, a WATERS Column Heater Module, a SHODEX SB-805 HQ column (8.0 × 300 mm, exclusion limit: 4000 kDa), a SHODEX SB 803 HQ (8.0 × 300 mm, exclusion limit: 100 kDa), a SHODEX SB-802.5 HQ column (8.0 × 300 mm, exclusion limit: 10 kDa) and a DynaPro NanoStar Dynamic Light Scattering detector (Wyatt, USA) with an infrared laser wavelength of 785.6 nm. DLS experiments were performed on FP solutions at a concentration of 2 mg/mL in 50 mM NaNO_3_ + 0.02 vol% NaN_3_ buffer. A volume of 150 µL of the FPs solutions was injected into the SEC columns using an aqueous mobile phase containing 50 mM NaNO_3_ and 0.02 vol% NaN_3_ at a flow rate of 1 mL/min. DLS data were analysed using the DYNAMICS 7.1 software (Wyatt) using a dn/dc value of 0.134.

### Fluorescent probes’ mobility measurements

Confocal Laser Scanning Microscopy (CLSM) was used to assess fluorescent probes’ mobility by Fluorescence Recovery After Photobleaching (FRAP). Experiments were performed using a Leica TCS SP8 confocal microscope (Leica Microsystems, Germany) equipped with 63× oil-immersion objective and 488 nm and 553 nm laser lines in a controlled temperature room (20 ± 2 °C). 60-µm-thick sections of the different samples were cut from polyethylene glycol-embedded fragments, thoroughly washed and incubated overnight in 0.01% (w/v) fluorescent probe solutions. Sections were then mounted in the probe solutions between slide and cover slip sealed with polish for microscopy analysis. Acousto-Optical Tunable Filters (AOTF) were set to collect fluorescence emission from 562 to 650 nm. Images with a size of 128 × 256 pixels were acquired using a ×4.5 zoom factor with a scan frequency of 1400 Hz. A circular region of interest (ROI1) with a diameter of 3 µm was selected for bleaching. This ROI1 was located in the xylem of the poplar sections and centred on the secondary wall of early wood cells. The fluorescence intensity in ROI1 was corrected using the fluorescence measured in three control ROIs located nearby, and normalised to get a value of 1 before bleaching and of 0 immediately after bleaching according to (Eq. 1), where *I*_t_ is the normalised fluorescence intensity of ROI1 at time *t*, *I*_0_ is the normalised intensity of ROI1 immediately after bleaching and *I*_pre_ is the normalised intensity of ROI1 before the bleaching step.

1 $$R\left( t \right) = \frac{{I_{\text{t}} - I_{ 0} }}{{I_{\text{pre}} - I_{ 0} }}$$

Ten scans were conducted every 0.051 s with the 553 nm laser line set to a power of 1% before the sample was bleached, as these conditions allowed observing mainly the probes and only little or no fluorescence from lignin. Then, 20 short pulses were applied to ROI1 every 0.051 s with both 488 nm and 553 nm laser lines set to 100% power. The total duration of the bleaching step (1 s) was short enough to consider that recovery during bleaching was negligible [36]. Fluorescence recovery was measured with the 553 nm laser line set to 1% power by taking 200 images with a 0.051-s time delay, then 300 images with a 2-s time delay for a total recovery measurement of approximatively 10 min. FRAP experiments were repeated 8 times on each sample, at different *XY* positions, and an average recovery curve was calculated for probes’ measurement analyses. As the diffusion was considered to occur mainly in the XY plan, a double exponential equation with four parameters *a*, *b*, c and *d* (Eq. 2) was used to model the averaged curve with a calculated coefficient of determination *R*^*2*^ of the fitness that was always above 0.99 (SigmaPlot 12.0, Systat Software, USA).

2 $$R\left( t \right) = a\left( {1 - e^{ - bt} } \right) + c\left( {1 - e^{ - dt} } \right)$$

The mobile fraction of the probe MF is equal to the plateau value obtained when fluorescent recovery remains unchanged. In mathematical term, MF corresponds to the fluorescence recovery when *t* → ∞, so it could be calculated as a simplification of Eq. 2 (Eq. 3).

3 $${\text{MF}} = a + c$$

### Data and statistical analysis

Enzymatic hydrolysis and wet chemistry analyses were carried out in triplicates and the results are expressed as mean ± standard deviation. MFs are expressed as mean ± standard deviation calculated from the average of 8 single recovery curves. An analysis of variance (ANOVA) was realised on the obtained experimental values followed by a Tukey test with a significance level of probability set at *p* < 0.05. Statistical analyses were performed using the SigmaPlot 12.0 software.

Full factorial experiments were performed using the Design-Expert 8.0 software (Stat-Ease, USA). MF experimental data (mean ± standard deviation) were computed with three principal factors (pretreatments, probes’ size and saccharification time) and all possible single and interaction *F*-values, which are ratios of the variations between the samples means and variations within the samples, were calculated, as well as their relative *p* values [24].

## Results and discussion

### Effect of pretreatments on saccharification

**Fig. 1:**
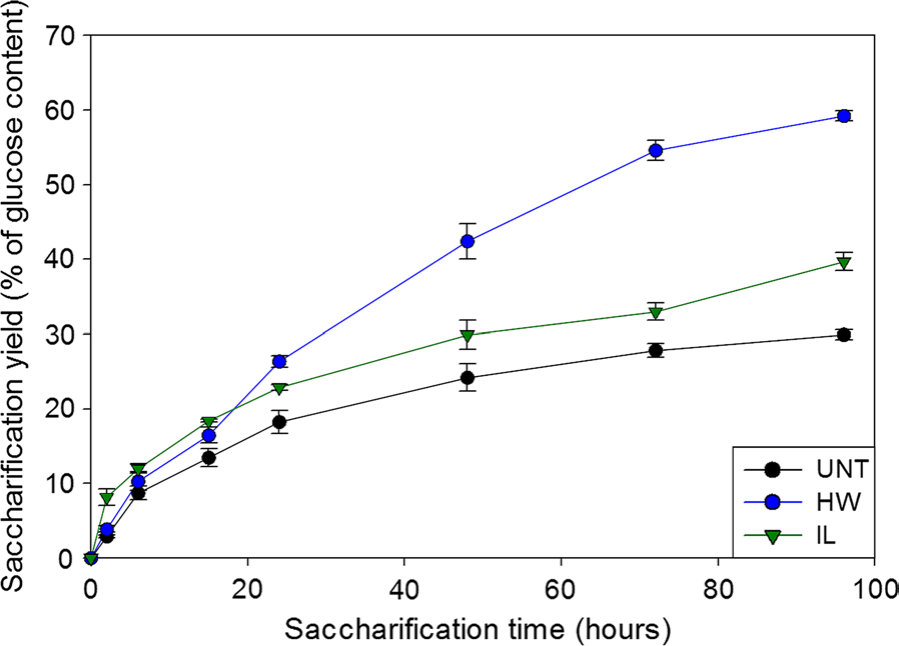
Kinetic release of glucose during enzymatic hydrolysis of the different poplar samples. Error bars indicate standard deviations. *UNT* untreated, *HW* hot water, *IL* ionic liquid

The contrasted effect of the pretreatments on poplar samples was first evidenced by the different sample weight losses of 32% and 9% after HW and IL pretreatments, respectively. Pretreatments efficiency was further investigated by carrying out saccharification experiments. Figure 1 displays the kinetic profiles of glucose release over the 96-h enzymatic hydrolysis of both untreated and pretreated poplar samples. Glucose was expressed as a percentage of the initial glucose content of the samples that was 43%, 52% and 46% of dry matter for the untreated, HW- and IL-pretreated poplar samples, respectively.

Untreated samples showed an increase in glucose release over time progressively slowing down after 6 h. The plateau of the curve was almost reached after 72 h of enzymatic hydrolysis with a final yield of 30% of the initial glucose content. Both HW and IL pretreatment allowed an improvement of the saccharification, but to different extents. Kinetics of HW-pretreated and untreated samples were similar for the first 6 h, but glucose release rate only slowed down after 72 h for the HW-pretreated sample, resulting in a final yield of 60% of the initial glucose content. For IL-pretreated samples, the glucose release during the first 2 h of reaction was around 2.5 times more important than what was observed with the two other samples. The reaction then slowed down and followed a similar trend to that of untreated samples. The final saccharification yield was 40% of the initial glucose content for IL-pretreated samples.

### Evolution of the chemical composition during the saccharification

**Fig. 2:**
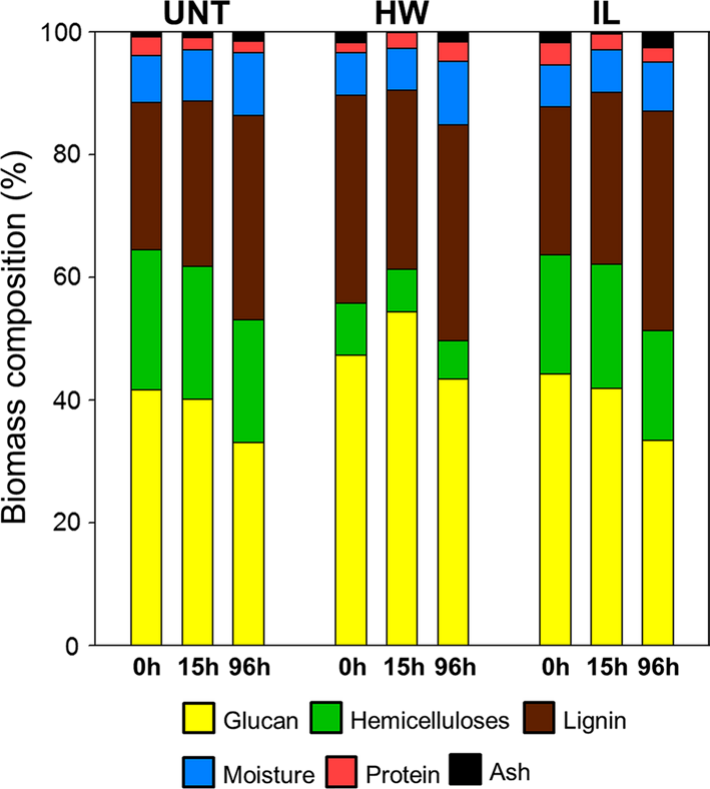
Dynamical assessment of the chemical composition of the different samples during saccharification. Contents are expressed as a percentage of dry matter. *UNT* untreated, *HW* hot water, *IL* ionic liquid

The overall chemical composition of the untreated and pretreated samples was assessed at different time points during the saccharification (Fig. 2). In addition to the initial (*t* = 0 h) and final time points (*t* = 96 h), an intermediary time point at 15 h was selected, as half of the glucose yield of the untreated samples was reached after 15 h (Fig. 1). The differences in glucose release between the untreated and both pretreated samples also started to become more important at 15 h (Fig. 1).

Both pretreatments induced an increase in glucan content from 41% in untreated samples to 47% (*p* < 0.001) and 44% (*p* = 0.002) in the HW- and IL-pretreated samples, respectively. HW pretreatment also induced a decrease of the hemicelluloses content from 22% down to 8% which resulted in an increase of the relative amount of lignin (from 24% up to 33%). IL pretreatment only induced a small decrease in hemicelluloses content down to 19% while the lignin content remained unchanged [13].

For the untreated samples, the relative glucan and hemicelluloses content remained similar during the first 15 h whereas the lignin content slightly increased (from 24 to 27%, *p* < 0.01). As half of the final amount of glucose was released during the first 15 h of hydrolysis (Fig. 1), it is likely that cellulose and hemicelluloses were degraded in the same proportion since the Cellic^®^ CTec 2 cocktail also contains some hemicellulases. After 96 h of saccharification, a decrease in the glucan content was observed (from 41 to 33%) and a smaller reduction of the hemicelluloses content was also quantified. On the contrary, the lignin content kept going up to 33%. This increase revealed that lignin was probably not or little affected by the enzymatic hydrolysis.

Similar observations could be made for the IL-pretreated samples, with the glucan content significantly decreasing only after 15 h (from 44 to 33%), while hemicelluloses content remained unchanged. Interestingly, the relative lignin content remained similar during the first 15 h of reaction (*p* = 0.072), whereas most of the glucose was released from the samples during this time (Fig. 1). This removal of lignin during the first 15 h of saccharification could be a consequence of some modifications of the lignin features. Previous analyses showed that the S/G ratio of poplar samples increased after IL pretreatment while the G units content decreased [13]. A relatively higher proportion of S units could make lignin more prone to partial degradation under the mild conditions of enzymatic hydrolysis, as S units are more likely to establish labile β-*O*-4′ linkages due to the steric hindrance of the methoxy groups on the aryl moieties [37].

HW-pretreated samples showed some differences in the evolution of their composition. The observed glucan content decrease after 96 h of hydrolysis (from 47 to 43%) was less important than that of IL-pretreated samples (from 44 to 33%). Unexpectedly, glucan content was even increased during the first 15 h (from 47 to 54%, *p* = 0.001), because of the reduction of the lignin content during the first 15 h of reaction, from 34 to 30% (*p* = 0.026). Indeed, the HW pretreatment caused a partial condensation of the lignin, which was partially aggregated to form small droplets that could be observed by electron microscopy [13]. It is likely that this reorganised lignin was removed from the samples as the cell wall polysaccharides, and especially the cellulose, were hydrolysed, causing a decrease of the relative lignin content and an apparent increase in the relative glucan content. This decrease in lignin content during the first 15 h might be the reason why the hydrolysis kinetics of the HW-pretreated samples retained a faster rate than those of untreated and IL-pretreated samples (Fig. 1). After 15 h of reaction, the evolution of the chemical composition became similar to what was previously described, with a decrease in glucan content and an increase in relative lignin content (from 30 to 35%, *p* = 0.018).

### Evolution of the accessibility during saccharification

**Fig. 3:**
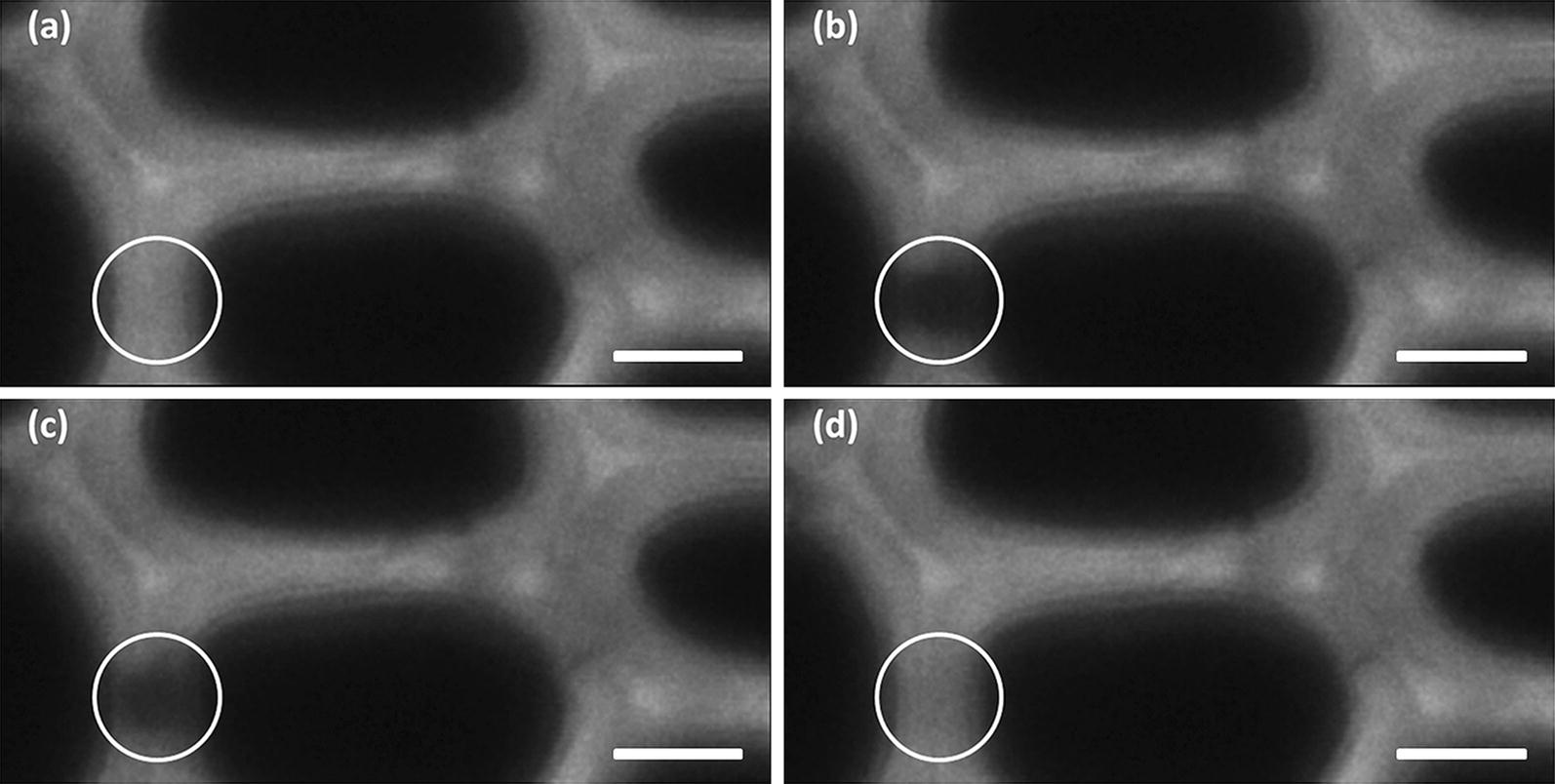
Visualisation of the course of a FRAP experiments. These images displayed the recovery of fluorescence of the 1.3-nm PEG-rhodamine probe within a ROI located on the cell wall of an IL-pretreated poplar section (dark area inside the white circle). **a** Before bleaching, **b** immediately after bleaching, **c** 10 s after bleaching, **d** 10 min after bleaching. Scale bar: 5 nm

To better understand how pretreatments impacted the saccharification efficiency, FRAP experiments were performed to study the mobility of PEG-rhodamine probes of different sizes within ROIs centred on the secondary cell walls of early wood cells (Fig. 3).

**Fig. 4:**
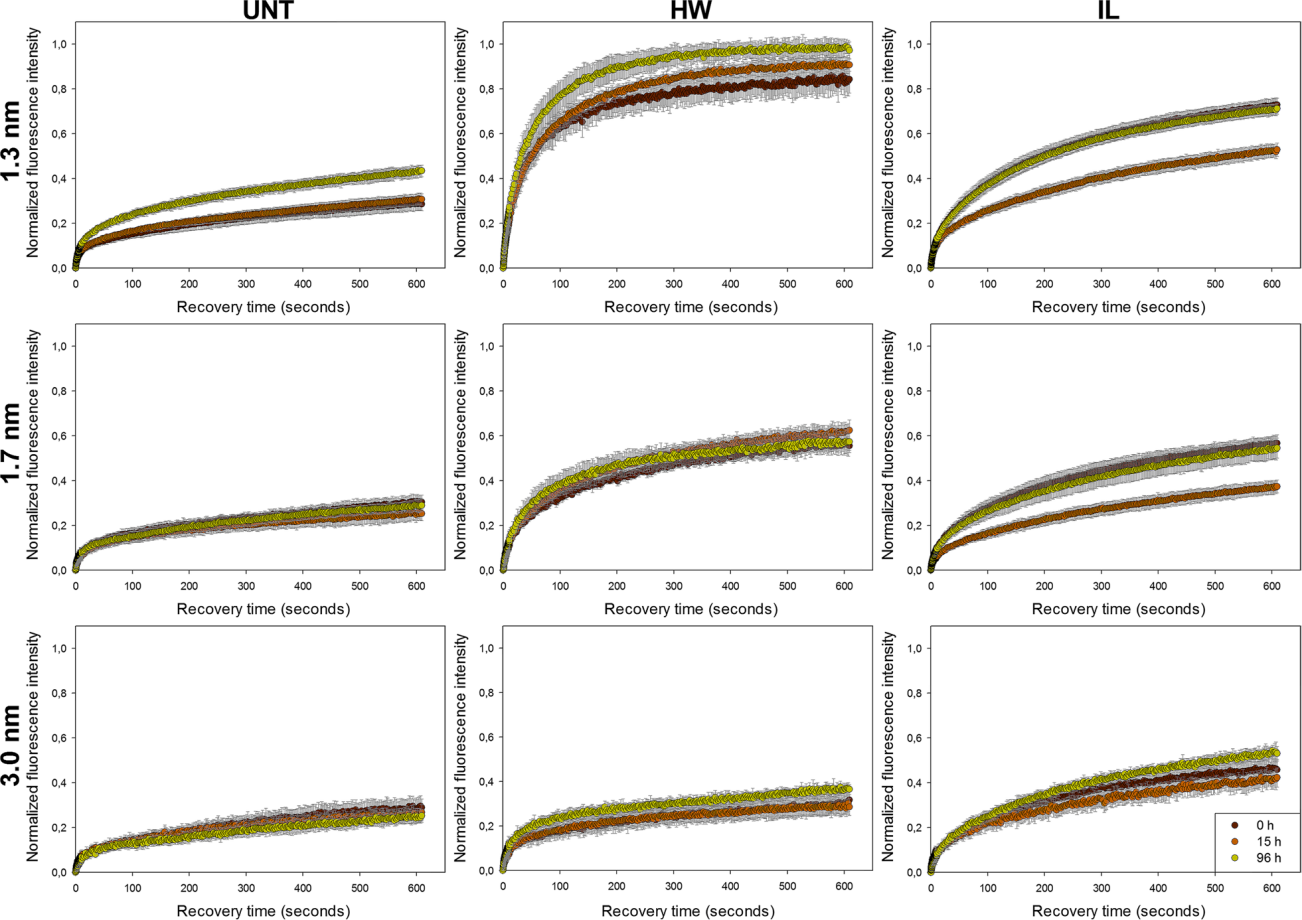
Fluorescence recovery curves of the different PEG-rhodamine probes measured on the untreated and pretreated samples enzymatically hydrolysed for 0 h (brown), 15 h (orange) and 96 h (green). Error bars represent the standard deviation between 8 recovery replicates. *UNT* untreated, *HW* hot water, *IL* ionic liquid

Three rhodamine-PEG probes were selected, with different hydrodynamic radii (*R*_H_) of 1.3 ± 0.5, 1.7 ± 0.2 and 3.0 ± 0.2 nm as determined by dynamic light scattering (DLS). These radius values are in the same range as those obtained using DLS on cellulolytic enzymes from different organisms [25, 38] and also with the nominal diameter of 5.1 nm which is generally admitted to be representative of the diameter of cellulases [39]. Accessibility of the three PEG-rhodamine probes was measured by FRAP in the untreated and pretreated samples at the three previously selected saccharification time points (0 h, 15 h and 96 h) (Fig. 4).

The recovery curves of the probes were very similar in all untreated samples, with the only exception of the 1.3-nm probes whose recovery was more important after 96 h of hydrolysis. In comparison, both HW and IL pretreatments induced an enhancement of fluorescence recovery for all probes, but fluorescence increased more quickly in the early phase of the recovery in HW-pretreated samples, suggesting a faster diffusion of the probes. Probes’ size also had an influence on fluorescence recovery for pretreated samples: the smaller the probe was, the higher the recovery was, with variations essentially depending on pretreatment. Effect of saccharification time was not essential and could only be deciphered for the 1.3-nm probe. It is noteworthy to mention that none of the recovery curves perfectly reached a plateau except the curves related to the diffusion of the 1.3-nm probes in the HW-pretreated samples. Equilibrium between bleached and non-bleached fluorescent probes was not reached yet after 10 min. Indeed, a two-phase diffusion was observed for all probes, with a fast initial recovery followed by a slowed down increase in fluorescence probably caused by interaction with lignin.

**Fig. 5:**
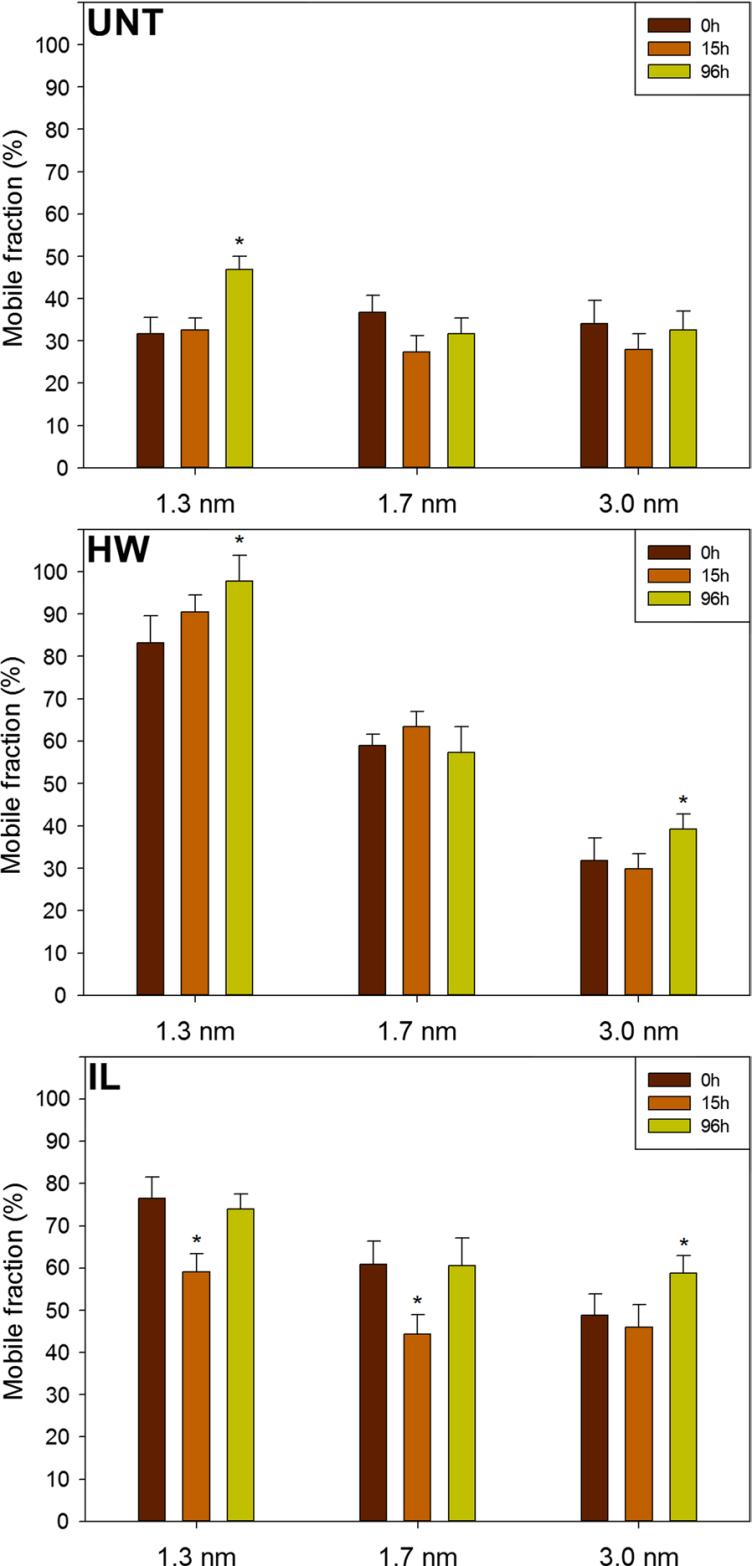
Mobile fractions of the different PEG-rhodamine probes measured in untreated and pretreated samples enzymatically hydrolysed for 0 h, 15 h and 96 h. Error bars represent the standard deviations of the mobile fraction calculated from the averaged recovery curve. Asterisks indicate statistically significant difference between the MF of a probe at a considered saccharification time point with its MF at 0 h. *UNT* untreated, *HW* hot water, *IL* ionic liquid

To evaluate quantitatively these differences, the experimental recovery curves were mathematically fitted to quantify the mobile fraction (MF) of the probes which is a measurement of the proportion of probes that can move freely inside the sample (Fig. 5).

Both HW and IL pretreatments induced an increase in MF of the different probes, but with different trends. While the MF of the 1.3-nm probe was more important after HW pretreatment than after IL pretreatment, both pretreatments allowed a similar increase in the MF of the 1.7-nm probe. Only the IL pretreatment allowed a significant MF increase (*p* < 0.001) of the 3.0-nm probe. Probes’ size seemed to have no influence on the MF in untreated samples as the MFs of the three probes were similar. After pretreatments, a decrease in MF could be observed as the probes’ sizes increased, going from 83% for the 1.3-nm probe to 32% for the 3.0-nm probe in the HW-pretreated samples and from 77% for the 1.3-nm probe to 49% for the 3.0-nm probe in the IL-pretreated samples.

The evolution of MF was also assessed during the saccharification for the different samples. In untreated samples, all probes displayed a similar MF at the different times of hydrolysis, apart from the 1.3-nm probe whose MF increased from 32% at 0 h and 15 h of saccharification to 47% after 96 h. Similarly, the 1.3-nm probe only displayed a significantly different MF after 96 h of hydrolysis (*p* = 0.042). The same trend was observed for the 3.0-nm probe in both pretreated samples, whereas no significant evolution of the MF was measured for the 1.7-nm probe. Surprisingly, the MFs of the 1.3-nm and 1.7-nm probes in the IL-pretreated samples were lower at 15 h compared to their respective measured values at 0 h and 96 h. These results showed the moderate influence of the saccharification time on accessibility, with no positive evolutions, while 30% and 50% of the final glucose concentration was already released after 15 h of hydrolysis for the HW-pretreated and untreated samples, respectively (Fig. 1). This emphasises that enzymatically catalysed degradation of plant cell walls’ polysaccharides induced small changes in probes accessibility. Pihlajaniemi et al. observed that porosity was not modified during the saccharification of pretreated wheat straw [40]. A possible explanation would be that the hydrolysis of cellulose and hemicelluloses could make room for the remaining lignin to expand, thus partially filling the gap left by the degraded polysaccharides and impeding tested probes and thus enzymes from diffusing more easily.

The evolution of the MF showed that diffusion in poplar cell walls was impacted by the modification in composition and structure induced by the pretreatment, the size of the probes but much less by saccharification time. To quantify the relative importance of each factor on the diffusion of the probes, a full factorial experiment was designed in which MF was considered as a response which depends on three different factors, namely the pretreatment (factor A), the probes’ size (B) and the saccharification time (C). Influence of factors considered alone and in interaction was assessed (Table 1).

**Table 1:** ANOVA analysis of the effect of pretreatment, probes’ size, saccharification time and their interaction on probes’ MF

Factors	*F*-value	*p*-values
A—pretreatment	303.02	< 0.0001
B—probes’ size	237.41	< 0.0001
C—time	23.83	< 0.0001
AB	76.15	< 0.0001
AC	6.94	0.0001
BC	3.69	0.0099
ABC	3.48	0.0027

The *p* values calculated for each factor or their interactions were all below 0.05, indicating that each factor considered separately and each interaction between factors had a significant influence on the diffusion of the probes

*F*-values indicate the contribution of the related factor to the MF: the higher the *F*-value is, the stronger the influence of the related factor is. As expected, pretreatment had the most important impact, followed by the probes’ size. Both had an *F*-value at least ten times higher than saccharification time. Considering factor interactions, only the combined influence of pretreatment and probes’ size presented a relatively high *F*-value, which makes sense since these factors have an important influence when considered alone. Overall, modifications induced by the HW and IL pretreatments on both composition and structure of the poplar cell walls were the main factors responsible for changes in probes’ mobility. Consequently, the size of the probes was also important, as it can influence the penetration into some pores depending on their diameter. The lower impact of saccharification time means that the removal of the polysaccharides during enzymatic hydrolysis resulted in minor modifications of the samples composition (Fig. 2) and structure compared to the effect of pretreatment. A recent study showed an increase in cellulose accessible surface area of diluted acid-pretreated poplar during the first 8 h of reaction then decreasing back to the initial value after 20 h of reaction [41]. Cellulose-accessible surface partially results from an increase in porosity of the cell wall. However, the fact that probes’ diffusion did not evolve after 15 h of reaction tends to show that porosity was not or only slightly improved during enzymatic hydrolysis.

### Porosity changes induced by the pretreatments

**Fig. 6:**
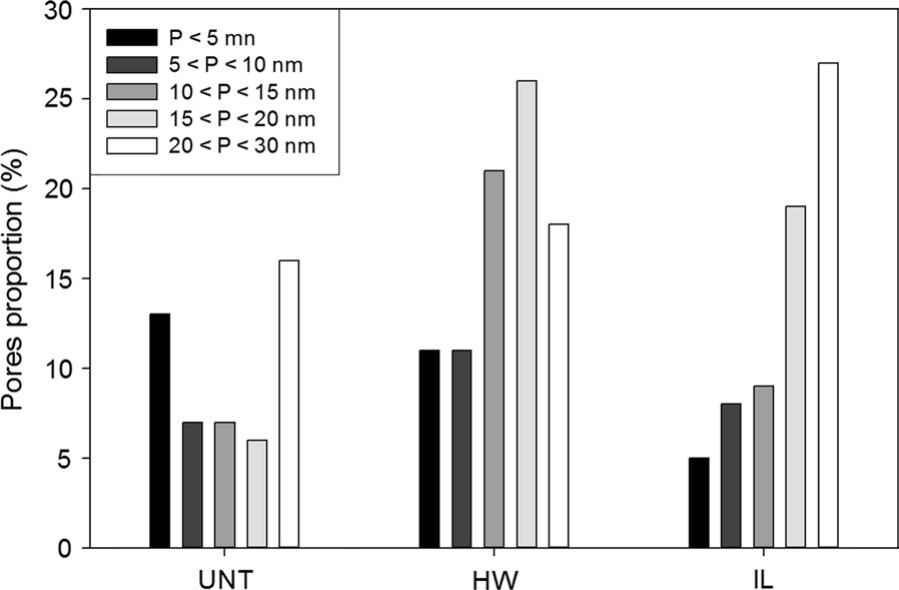
Distribution of pores’ size ranges in the untreated and pretreated samples. Pores proportions are expressed as a percentage of the total porosity of the samples, so that the sum of the proportions is below 100% since only pores below 30 nm are considered. *UNT* untreated, *HW* hot water, *IL* ionic liquid

To better understand the changes of probes’ mobility observed previously, the porosity of the samples was also investigated since it is likely to influence enzymes’ diffusion in the plant cell walls [42]. As enzymatic hydrolysis was found to have a low impact on mobility compared to pretreatment, only the porosity of untreated and pretreated samples before saccharification was analysed. NMR analysis of the relaxation time of water absorbed within the samples was used to determine samples porosity. In comparison to other techniques such as nitrogen adsorption of mercury porosimetry, samples did not require any drying step that might be responsible for a collapsing of the pores [18]. Rather, samples are soaked in water so that the environment is close to that of enzymatic hydrolysis. Although the overall porosity of the samples was measured, only pores with a diameter below 30 nm were taken into account (Fig. 6), as these are the most likely to influence probes mobility into the plant cell walls based on their size.

Untreated samples displayed a relatively high proportion of pores with a diameter below 5 nm (13%). The pores ranges comprised between 5 and 20 nm had lower proportion around 6–7% while pores in the range 20–30 nm were the most important representing 16% of the total porosity. Altogether, these pores ranges accounted for 49% of the overall porosity of untreated samples.

Porosity below 30 nm increased after the different pretreatments, reaching 87% and 68% for the HW-pretreated and IL-pretreated samples, respectively. This rise was not due to the pores with a diameter below 5 nm, whose proportions were the only ones to decrease among all the measured ranges (11% and 5% for the HW-pretreated and IL-pretreated samples, respectively). In HW-pretreated samples, all other pore size ranges increased, most notably the pores in the range 10–15 nm (threefold) and 15–20 nm (4.3-fold). In IL-pretreated samples, the increase in the proportion of pores with a diameter comprised between 5 and 15 nm was less important (proportion of 14% and 17% for the untreated and IL-treated samples, respectively). As with the HW-pretreated samples, the highest augmentation was observed for the pores in the range 15–20 nm with an increase by 3.2 times compared to the untreated samples. The most important range measured was the 20–30-nm pores which represented 27% of the overall porosity.

The increase in porosity observed after both pretreatments is likely to be at least partially responsible for the increase in glucose release during the saccharification of the pretreated samples. Indeed, saccharification and global porosity followed the same order regarding pretreatment: HW > IL > UNT. More precisely, the most important increase in pores was related to the range 10–20 nm for both pretreatments, indicating their major influence for an effective diffusion of lignocellulolytic enzymes. These results are in agreement with the assumption that a pore diameter of 5–10 nm is too small to allow a significant diffusion of enzymes [39]. Recently, Hinkle et al. showed by electron tomography that the increase in saccharification efficiency induced by steam-explosion pretreatment of corn stover resulted from an increase in porosity and thus in accessible surface area for enzymes below a threshold of roughly 5–10-nm radius [23]. An increase in the proportion of nanopores with a diameter in the range 10–100 nm formed between microfibrils during dilute acid and HW pretreatment of samples was considered as the most fundamental barrier to overcome to allow an efficient enzymatic hydrolysis [43]. Larger pores with diameters over 20 nm might be non-essential for an effective saccharification as their proportion was more important in IL-pretreated samples than in HW-pretreated samples.

### Influence of lignin on accessibility

The MF of PEG-rhodamine probes within the plant cell walls can be impacted by both cell wall’s porosity and interaction with accessible lignin. Considering these features, only porosity was largely shifted by pretreatments (Fig. 6), lignin content was only moderately affected (Fig. 2). However, porosity alone could not explain the increased accessibility of the probes: for example, after pretreatment, the MF of the 3.0-nm probe in the IL-pretreated samples was the highest, whereas HW-pretreated samples displayed a higher amount of pores with a diameter below 20 nm. In addition, the diffusion of this probe was the fastest in the HW-pretreated samples at the beginning of the recovery compared to the other samples, as the fluorescence increased more rapidly (Fig. 4). The fact that recovery was then slowed down could be related not only to lignin content, but also to lignin structure. NMR and thioacidolysis reaction analyses of the same samples both showed that lignin underwent condensation reaction after HW pretreatment, whereas it was only slightly modified after IL pretreatment [13]. This condensation might be responsible for the less important diffusion of the largest probes in the secondary part of the recovery compared to that in IL-pretreated samples, as studies showed that enzymes are more prone to bind lignin that has underwent condensation reactions [44, 45]. A recent report also showed using confocal microscopy approaches that lignin accessible surface increased while cellulose surface decreased during saccharification of raw poplar samples, making cell wall surface less prone to degradation through the course of enzymatic hydrolysis [46]. An increase of lignin accessible surface is likely to favour non-productive interactions of enzymes with lignin. These interactions are thought to occur mainly through hydrophobic interactions as well as hydrogen bonding, depending on the characteristics of the enzymes [47]. FRAP experiments could then be designed to better understand the dynamic behaviour of isolated enzymes in plant cell walls, and more especially their interactions with both cellulose and lignin. Overall, the evolution of the probes’ accessibility during saccharification is likely not related to modifications of the lignin content but rather to lignin structural and chemical modifications.

### Correlations between saccharification and accessibility

**Fig. 7:**
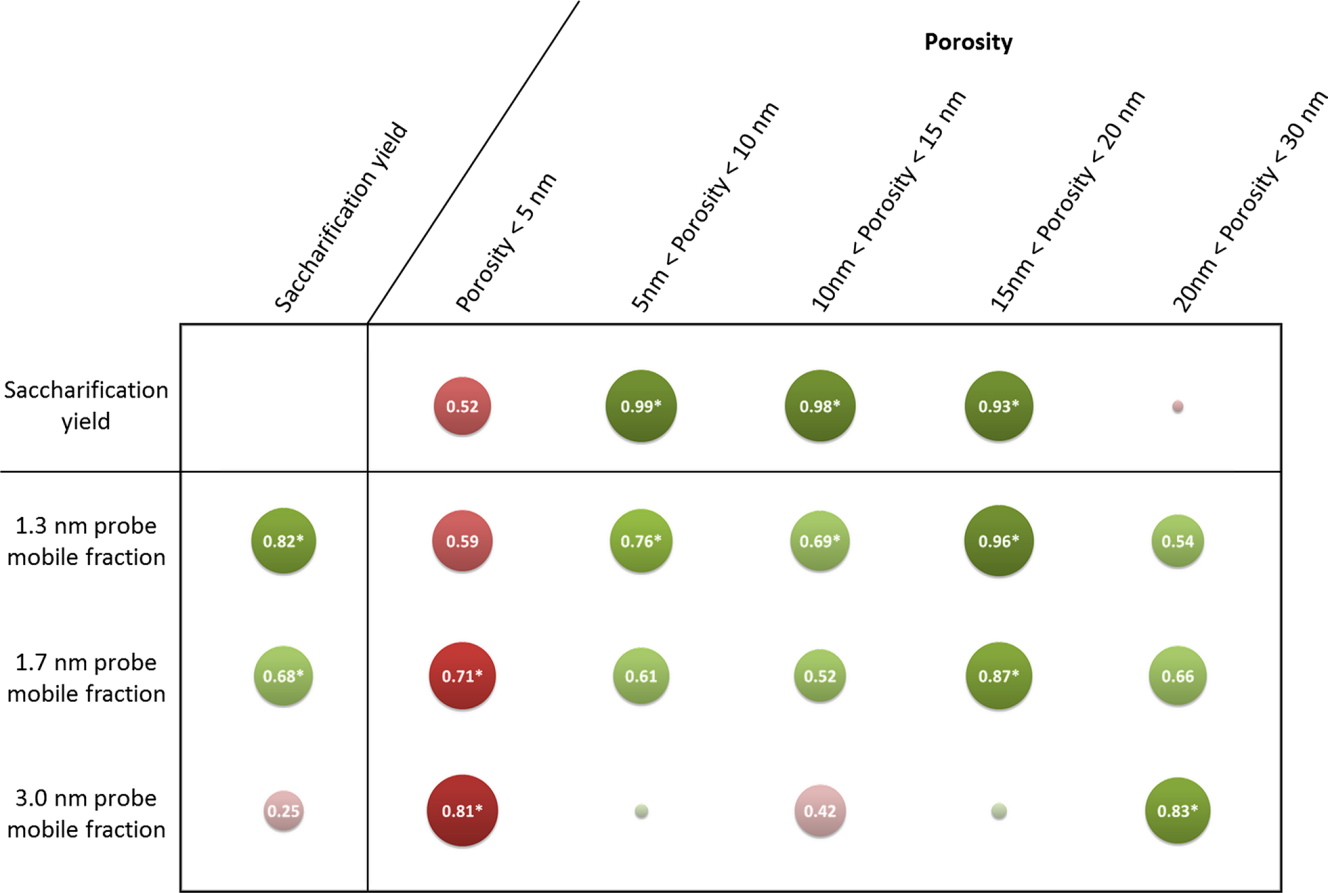
Pearson’s correlations coefficients between saccharification yields, probes’ MF and samples porosity. Discs diameter and colour shades display the strength of the correlations. Both positive and negative correlations are presented using the absolute value of the coefficients. Absolute values under 0.1 are not indicated on the corresponding discs. Positive and negative correlations are displayed in green and red, respectively. Asterisks indicate statistically significant coefficients with a *p* value below 0.05

The results obtained on untreated and pretreated samples showed that probes’ mobility was related to samples porosity measured before saccharification, and also to the final sample saccharification yield. Pearson’s correlations coefficients were calculated to investigate in details the previously made assumptions (Fig. 7).

An evolution of the correlations with porosity was observed according to the probes’ size. The best correlations were observed for the 1.3-nm and 1.7-nm probes with the ranges 15–20 nm, and with the range 20–30 nm for the 3.0-nm probe. The fact that probes diffuse better in larger pores as their size increases makes sense. Interestingly, the best correlations were not observed with the porosity range corresponding to probes’ diameter. For porosity to allow a significant enhancement of PEG probes’ diffusion, pores diameter had to be around five times the diameter (or ten times the *R*_H_) of the probes. This might be explained by the interactions of PEG with lignin, meaning that probes are likely to fill in the pores as they get bound to the lignin, hampering the diffusion of other probes if the initial pore diameter is too small.

Saccharification yield displayed strong significant correlations with MFs of the 1.3-nm and 1.7-nm probes, with coefficients values of 0.82 and 0.68, respectively. No statistically significant correlation was observed between the final glucose yield and MF of the 3.0-nm probe as its diffusion was more important in IL-pretreated samples compared to HW-pretreated samples (Fig. 5), whereas the latter sample was the most efficiently hydrolysed (Fig. 1). The fact that saccharification yield could not be related to the mobility of all probes showed that enzymatic hydrolysis does not depend solely on enzymes’ diffusion. Saccharification efficiency has been shown to be highly dependent on cellulose accessibility [48]. Increase in pore volumes always leads to an increase in accessible surface area [6]. However, for a considered volume, a higher proportion of small pores as observed in the HW-pretreated samples would result in a higher accessible surface than larger pores as the one observed after IL pretreatment. Jeoh et al. demonstrated using fluorescence microscopy techniques that improving porosity may have little or no impact on biomass digestibility unless cellulose is made more accessible to enzymes [49]. For enzymes’ diffusion and mobility to be correlated, a compromise in pores diameter increase must be obtained: pores have to be large enough to let enzymes go through, but not too large so that accessible surface area remains adequate.

## Conclusions

Confocal microscopy was combined to porosity measurements and chemical analysis to get a better understanding of how porosity and chemical composition impact enzymes’ diffusion and activity within poplar cell walls. Probes’ diffusion depended on their size and was mainly influenced by the changes in the structural and chemical composition of the samples induced by pretreatments. Saccharification caused slight changes in accessibility only after 96 h of reaction in a few cases, which means that the cell wall’s network was not extensively modified during saccharification, probably because of the rearrangement of lignin residues. Lignin, whose relative content was increased during the saccharification, possibly underwent some changes in its spatial conformation impeding probes’ diffusion by filling the gap left by the hydrolysed polysaccharides.

Improvements in porosity allowed a better diffusion of the probes. The best correlations between probes’ mobility and porosity ranges were obtained for pores with a diameter at least five times the size of the probes. Pores in the range 5–20 nm governed saccharification in our samples, as their proportion was strongly correlated with saccharification yield, while bigger pores had a detrimental effect. Overall, to improve saccharification, an increase in pore size is necessary to allow a better accessibility while limiting interaction with lignin but it has to be moderate, probably to maintain a sufficient accessible surface area. The influence of interactions with lignin could be further studied by applying more sophisticated recovery model to fit the FRAP recovery curves that would give more information on both the diffusion and bonding kinetics. Those equations would require a better understanding on the nature and distribution of the binding site [50].

## Abbreviations

AOTF: acousto-optic tunable filter
CLSM: confocal laser scanning microscopy
DLS: dynamic light scattering
FRAP: fluorescence recovery after photobleaching
HPAEC–PAD: high-performance anion-exchange chromatography with pulsed amperometric detection
HW: hot water
IL: ionic liquid
LF-NMR: low-field nuclear magnetic resonance
PEG: polyethylene glycol
*R*_H_: hydrodynamic radius
ROI: region of interest
